# Using a Studio-Academic Partnership to Advance Public Health Within a Pragmatic Yoga Setting

**DOI:** 10.1177/2150132719874621

**Published:** 2019-09-20

**Authors:** Samantha M. Harden, Abby M. Steketee, Rachel Kelliher, Keala A. Mason, Nicole Fitzwater Boyle

**Affiliations:** 1 Virginia Tech, Blacksburg, VA, USA; 2 In Balance Yoga Studio, Blacksburg, VA, USA

**Keywords:** cohesion, community-based, participatory approaches, translation

## Abstract

**Objectives:** To explore community-based yoga studio practitioners’ psychosocial variables, behaviors, and studio satisfaction. **Methods:** Concurrent mixed-methods study consisted of a survey for demographic variables and psychosocial variables of interest (e.g., mindfulness, self-compassion, physical activity participation) and interviews regarding reasons for participating at the yoga studio. **Results:** Participants (N = 138) were, on average, 35.58 ± 14.09 years old and predominantly female (91.3%), married (40.6%) or single (37%), Caucasian (75%), and college (25.4%) or graduate/medical school (45%) educated, with 54% meeting physical activity recommendations. On a 5-point Likert-type scale, participants reported being moderately cohesive (*M*_sumscore_ = 3.87 ± 0.62), stressed (*M*_sumscore_ = 3.2 ± 0.39), mindful (*M*_sumscore_ = 3.4 ± 0.41), and self-compassionate (*M*_sumscore_ = 3.26 ± 0.56). A rapid content analysis of interviews (n = 18), indicated that participants primarily practiced at the studio for the sense of community. **Conclusions:** Yoga practitioners reported positive perceptions and behaviors; however, opportunities remain for interventions to improve mental and physical health among individuals already attending a yoga studio. Through an academic-studio partnership, studio offerings may include low-dose evidence-based interventions to improve access to and uptake of a yoga practice.

## Introduction

We need yoga more now (than ever) . . . human beings have always needed self-inquiry and presence and steadiness of mind . . . but because of the pace of our culture, the loneliness in our culture, how we live . . . this is a new phenomenon in the history of human beings . . . We have a lot of stress. We do not go out and work in the field . . . or go on a hunt . . . When people come to a yoga class, they have been very likely sitting too much, not eating well, drinking too much coffee. . . . trying to do too many things . . . When we have a yoga class, we are creating, whether we know it or not, community,

states renowned yoga instructor, author, and physical therapist Judith Hanson Lasater.^1^ Within this quote, public health practitioners and researchers can identify complex, interrelated health concerns such as stress, isolation, and inactivity—all of which may be mitigated through the practice of yoga.^2-4^

Studying the practice of yoga as a public health intervention^2,5^ is of particular interest for a number of reasons. First, 21 million Americans practiced yoga at least once in the past year.^6^ Second, because most yoga studies are epidemiological, cross-sectional, or based on efficacy trials with explanatory participant samples,^3,4,7-9^ there is low generalizability and pragmaticism—that is, the ability of the evidence-base of yoga to translate into the real world.^10^ Third, many modern versions of yoga are not captured in yoga research, which has focused on clinical populations in controlled settings performing traditional styles of yoga such as hatha, Iyengar, and ashtanga.^11^ This may limit the match of the evidence-base for health-enhancing yoga benefits and real-world practice. Finally, there is a call for more context cognizant scholarship, moving beyond proof of concept yoga interventions to understanding real world yoga practices and adherence (including an individual’s regular practice, not adherence to an evidence-based intervention).^12^

Therefore, there is a prime opportunity to understand the behaviors and health factors of individuals within settings where people are naturally attending yoga classes: Their community-based yoga studio. There are over 6000 yoga studios nationwide and yoga represents a $16 billion industry.^13^ Yoga studio owners have a vested interest in understanding how to keep clients engaged, and public health investigators are interested in matching evidence-based interventions with target audiences. All of this underscores the potential for an academic-community partnership^14^ to explore psychosocial variables, behaviors, and perceptions of yoga practitioners. Results identified in this exploratory study indicate areas for improvement—in terms of studio offerings and health promotion—as well as what is already working. Notably, for the purposes of this work, yoga is defined as a combination of breathing exercises (*pranayama*), meditation (*dhyana*), and postures (*asana*).

## Methods

### Setting

The college town of Blacksburg, Virginia is home to 42 000 residents of which 46% are female and 78% are Caucasian. The town has a large proportion of individuals who are 18 to 64 years old (82%), with only 13% and 5% of the population being younger than 18 or older than 65 years, respectively.^15^ This town is located in a southern state, a region of the nation with the lowest rates of yoga participation.^6^

At the time of the study, the community had 3 stand-alone yoga studios, and yoga classes at local gyms and through on-campus recreation. One studio of interest was the In Balance Yoga studio. The mission of the studio is to

support and lead a yoga community that practices and provides a variety of yoga to all populations and is passionate about giving back to each other and our planet . . . to progress and expand our community we offer an array of studio classes, trainings, workshops, and retreats within our yoga studio and globally.

The studio has a social entrepreneurship business model, which includes quarterly contributions to 3 nonprofit organizations. The key values of the owner and management are “positivity, service, connection, and fun.” In alignment with this mission and values, the studio engages in a number of strategies to enhance perceptions of cohesion/inclusivity, to reach and retain more community members, and to provide an environment that facilitates a consistent yoga practice.

Examples of these strategies include hosting approximately 47 external/guest instructors or teachers per year. This provides practitioners in rural, Southwest Virginia with a diverse group of experts from different yoga lineages and a balance from physical to spiritual expertise. Another example is that In Balance Yoga Studio offers annual studio-centric engagement challenges. For three years, there was a 30-day challenge. Program components included salient strategies for behavior change^16^: (1) goal setting and friendly competition to attend 30 classes in 30 days, (2) self-monitoring on a physical board in the studio space, and (3) feedback and auditing through recognition on social media and the October e-newsletter. Approximately 70 clients participated each year, for 3 years. External recognition of these efforts is seen in the studio’s receipt of the runner up for the wellness category of the “Best of the Blue Ridge” competition spanning from Maryland through Georgia along the East coast in the United States.

Knowing the values and mission of the studio, members of the research team approached the studio to discuss a potential partnership to explore psychological factors, attendance, and exercise among studio participants. Notably, the studio owner declined similar partnership offers from other researchers in the past. However, the studio owner perceived this research team to have buy-in and support of the studio and the desire to *partner* rather than simply access data.^14^ In addition, all members of the research team were also registered yoga instructors, 2 of whom were instructors at the partnering studio. Therefore, the owner accepted the invitation to explore these empirical and pragmatic outcomes of interest.

### Recruitment

Researchers recruited a convenience sample of yoga class attendees in a single community-based studio in rural Southwest Virginia. Participants were recruited through flyers posted at the studio, 2 posts to each the studio’s Facebook and Instagram accounts, and announcements in the studio’s e-newsletter (N = 1400 recipients). Finally, and in alignment with the mission for cruelty-free eating, vegan food was available from 9 am to 9 pm on a Sunday in October at the studio with members of the research team available to discuss the study. There are 8 classes every Sunday and there are typically 130 practitioners across a variety of class types (e.g., hot yoga, restorative, prenatal). The study was open to any individual who received the newsletter or attended yoga class at the studio. Study participants were entered in a prize drawing to receive one of two $50 studio gift certificates. The Virginia Tech Institutional Review Board approved this study.

### Research Design

This study used a concurrent, mixed-methods design.^17^ Cross-sectional surveys were provided for a 4-week period in online and paper-and-pencil formats. For the qualitative portion of the study, individuals attending the yoga studio during a research team–sponsored community event (where vegan food was offered) were randomly selected and asked if they would like to participate in a video-recorded testimonial. Once written consent was obtained, the individuals who agreed to participate in the study were interviewed by a researcher with one prompt: “Why do you practice at this studio?”

There are several indicators that can be used as the denominator for this study: total number of individuals who have attended class in the past 3 months (N = 10 951); total number of individuals who subscribe to the newsletter (N = 1400); and total number of individuals who attended class during the community event (N = 133).

### Quantitative

#### Measures

##### Attendance

The studio uses the MindBody business software to develop practitioner accounts and track studio attendance. With consent, practitioners’ attendance data were pulled from their accounts for the previous 12 months. These data were used to develop practice indicators of (1) total number of studio classes in the past 12 months, (2) average number of studio classes per week in the past 12 months, and (3) duration of membership.

##### Cohesion

A modified version of the 21-item Physical Activity Group Environment Questionnaire^18^ was employed. To be consistent with all other scales in the study, the items were converted from a 9-point Likert-type scale to a 5-point Likert-type scale. Items were modified from “I like the amount of physical activity I get with this group” to “I like the amount of physical activity I get from yoga classes here.”

##### Physical activity

The Stanford Leisure-Time Activity Categorical Item (L-Cat)^19^ is a single item survey with 6 descriptive categories ranging from inactive to very active, which was shown to have strong psychometric properties with high validity in overweight/obese women. According to the interpretation guide, if participants indicated a category 4 or higher, they were coded as meeting the physical activity guidelines for Americans (1 = yes, 0 = no).

##### Perceived stress

The validated, 10-item Perceived Stress Scale (PSS)^20^ was used to assess stress. Each item is on a 5-point Likert-type scale (1 = never, 5 = very often) and an example item is “In the past month, how often have you felt that you were unable to control important things in your life?” PSS scores are obtained by reverse-coding responses to the 4 positively stated items (items 4, 5, 7, and 8) and subsequently summing across all scale items. The higher the overall score, the higher the perceived stress.

##### Self-compassion

The validated, 12-item Self-Compassion Short form^21^ was used to assess the degree to which participants demonstrate self-compassion as defined as emotional regulation to appraise painful or distressing feelings with kindness, understanding, and sense of shared humanity, instead of harshly judging oneself or ignoring distressing feelings. All items were on a 5-point Likert-type scale (1 = never, 5 = very often) and example items include, “When I’m feeling down, I tend to obsess and fixate on everything that’s wrong.” Self-compassion scores were obtained by reverse-coding responses that indicate an uncompassionate thought, feeling, or action. The higher the overall score, the higher the level of self-compassion.

##### Mindfulness

The 24-item Five Facet Mindfulness Questionnaire: Short Form^22^ was used to assess (1) nonreactivity to inner experience, (2) observing/noticing/attending to sensations/perceptions/thoughts/feelings, (3) acting with awareness/automatic pilot/concentration/nondistraction, (4) describing/labeling with words, (5) nonjudging of experience (Baer et al., 2006).^23^ Each item is on a 5-point Likert-type scale (1 = never or very rarely true, 5 = very often or always true). An example item is “I do jobs or tasks automatically without being aware of what I’m doing.” Following the scoring recommendations, where appropriate, items were reverse coded, and the sum scores were used in analysis. The higher the overall score, the higher the level of mindfulness.

##### Studio perceptions

These items were developed in partnership with the studio representatives. The items were related to cleanliness, trust, class offerings, and the environment. To match the scales in the rest of the survey, all items were on a 5-point Likert-type scale (1 = very strongly disagree, 5 = very strongly agree). Example items include “I feel like this studio offers services/classes that meet the needs of all age ranges” and “The environment of this studio is calm and soothing.” These items, although not validated, were chosen to help understand “what matters” to the studio management.

### Analytical Plan

Quantitative data were descriptively summarized by proportions and means. Pearson correlation was used to detect associations between the psychometric variables in this study and membership status (number of classes attended), sex, teacher status, and each psychosocial variable (mindfulness, cohesion, stress, self-compassion). One-way analysis of variance was conducted on years of membership and age with the key outcomes of interest. The a priori hypotheses were that duration of membership, number of classes attended, female sex, and teacher status would lead to greater perceptions of cohesion, stronger mindfulness and self-compassion ratings, and lower perceived stress.

Following the completion of the community event, qualitative data were transcribed verbatim by a trained undergraduate research assistant using Microsoft Word. The names of the individuals who participated in the testimonials, in addition to any sensitive information (name of teachers, occupation, name of studio, etc) that they provided in their responses, were excluded from the transcription. In order to protect the identity of the participants, coded labels were assigned to each participant and used throughout the transcription process. The same undergraduate assistant performed a content analysis^24^ on the transcribed videos to identify the meaning units. Meaning units were any word or phrase that represented a single idea. Both the transcription and meaning units were submitted to the lead author for content review. Following this review, the meaning units were categorized into themes. Any meaning that was conveyed by 8 or more participants was operationalized as a major emergent theme whereas a meaning unit that was mentioned by 7 or fewer participants was considered a minor emergent theme.^25^ Notably, studio instructors and students were invited to provide testimonials, but the position of the participants at the yoga studio was disregarded in the content analysis of the transcriptions.

## Results

### Sample

Participants (n = 138) were aged 35.58 ± 14.09 years (range 19-72 years), predominantly female (91.3%), Caucasian (75%), and college (25.4%) or graduate/medical school (45%) educated. In addition, many participants (29%) had been practicing yoga for 4 to 7 years, and 10% were certified instructors at the studio. The sociodemographic characteristics of the sample are shown in Table 1. Thirty-one individuals were approached to provide testimonials, and 18 provided data (58%). A majority of invited yoga practitioners declined to provide a testimonial because of not wanting to be on film or being sweaty from class (or a combination of the two). Other unique reasons for declining the invitation for a video recorded testimonial were that they had not been a member of the studio long enough (in their opinion), that they had suffered a recent loss in their family, or that they did not have time to provide a testimonial. Three of the testimonials (17%) were provided by instructors at the yoga studio that volunteered and 83% (n = 15) were students at the yoga studio.

**Table 1.** Summary of Sample Characteristics and Outcomes From Key Variables.^a^

Demographic Characteristics		
Age, years (n = 125)	35.6 (±14)	
Race/Ethnicity (n = 129)		
Caucasian	108 (78.3)	
Asian	10 (7.75)	
African American	3 (2.2)	
Other	7 (5.1)	
Alaska Native	1 (0.7)	
Prefer not to answer	1 (0.7)	
Sex (n = 126)		
Male	12 (9.5)	
Female	114 (90.5)	
Education (n = 126)		
Grade 12 or GED	3 (2.4)	
College, 1-3 years	26 (20.2)	
College graduate	35 (27.8)	
Graduate/Medical school	62 (49.2)	
Yoga teacher status (n = 117)		
Not a teacher	61 (52.1)	
No, but would like to be a teacher someday	31 (26.5)	
Yes, 200 hours registered or higher	20 (17.1)	
Yes, not certified	5 (4.3)	
Distance from studio (n = 123)		
≤10 minutes	74 (60.1)	
11-20 minutes	37 (30.0)	
21-30 minutes	3 (2.4)	
31-40 minutes	2 (1.6)	
41-50 minutes	3 (2.4)	
51-60 minutes	1 (0.8)	
240 minutes	2 (1.6)	
Varies	1 (0.8)	
Behaviors (n = 106)		
Meeting physical activity recommendations	75 (54)	
Yoga practice/Studio specific		
Duration of membership in years	3.08 (±2.69)	
Number of classes attended past 12 months	56.94 (±60.36)	
Class most frequently attended (n = 116)		
Hot flow-set	32 (27.6)	
Warm flow	19 (16.4)	
Flow	3 (9.5)	
Donations	7 (6.0)	
Basic flow	6 (5.2)	
Power flow	5 (4.3)	
Gentle yoga	5 (4.3)	
Yin	5 (4.3)	
Other/Not listed	5 (4.3)	
Hot Flow Mix	4 (3.4)	
Demographic Characteristics		
Hot Flow2-Set	3 (2.6)	
Hot 26&2-Set	3 (2.6)	
Yoga Basics	2 (1.7)	
Prenatal yoga	2 (1.7)	
Aerial	2 (1.7)	
Restorative hammock yoga and meditation	2 (1.7)	
Meditation	1 (0.9)	
Donation/Community/Free class	1 (0.9)	
Pilates Sculpt	1 (0.9)	
Studio satisfaction (n = 116) on 5-point scale		
Studio satisfaction	4.53 (±0.67)	
Key Scale Sum Scores on 5-Point Scales	Full Sample	Mind-Body Approved
Cohesion (n = 106)	3.87 (±0.62)	3.89 (±0.59)
Perceived stress (n = 106)	3.20 (±0.39)	3.21 (±0.39)
Self-compassion (n = 109)	3.27 (±0.56)	3.23 (±0.52)
Mindfulness (n = 112)	3.42 (±0.41)	3.43 (±0.37)

a All data reported as means (±standard deviation) or number (percent).

### Quantitative

Of the 138 participants, 116 answered the question “What class do you attend the most (select one)?” Of the 29 class options, the top 3 most attended classes were Hot Flow-Set (23.2%), Warm Flow (13.8%), and Flow (8.0%). The least attended classes were Meditation (0.7%), Donations/Free Community (0.7%), and Pilates Sculpt (0.7%).

Of the 138 participants, 116 also answered the question “What classes do you typically attend at this studio (select all that apply)?” Of the 29 class options, the top 3 classes typically attended were Hot Flow-Set (49.3%), Warm Flow (43.5%), and Hot Flow Mix (31.9%). The 3 classes least typically attended were Restorative hammock yoga and meditation (2.2%), Fascae Freedom (0.7%), and Yoga for Seniors (0.7%). Based on a score of 4 or higher on the L-Cat, 54% (n = 75) of participants met physical activity recommendations.

Sum scores of the scales indicate that participants were sometimes stressed (*M*_sumscore_ = 3.2 ± 0.39), often mindful (*M*_sumscore_ = 3.4 ± 0.41), moderately self-compassionate (*M*_sumscore_ = 3.26 ± 0.56), and moderately cohesive (*M*_sumscore_ = 3.87 ± 0.62).

There was a significant relationship between duration of membership and number of days attended in the past year (*P* = .008) and mindfulness (*P* = .04). There was also a significant relationship between age and the days attended in the past year (*P* = .025) and duration of membership (*P* = .04). No other significant relationships were found between duration of membership or age with other key variables of interest (*P* > .05). Please see Table 2 for a full correlation matrix.

**Table 2.** Correlation Matrix for Psychosocial Variables and Attendance.

	M (SD), N (%)	1	2	3	4	5	6	7	8
Female sex (1)	80 (87)	—							
Yoga instructor status, affirmative (2)	14 (15)	−.04	—						
Number of days attended past 12 months^a^ (3)	59.94 (60.36)	−.11	.15	—					
Meeting physical activity recommendations (L-Cat) (4)	75 (54)	−.14	.16	.05	—				
PAGEQ sum score (5)	3.88 (0.59)	−.10	.14	.43**	−.00	—			
Self-compassion sum score (6)	3.22 (0.52)	−.18	.05	.31**	.02	.13	—		
Mindfulness sum score (7)	3.43 (0.37)	−.18	.09	.12	.02	.13	.61**	—	
Perceived stress sum score (8)	3.2 (0.39)	.17	.00	−.09	.02	−.18	−.31**	−.10	—

a Attendance and duration of membership are only with the N = 92 who provided access to MindBody data.

* Correlation is significant at the .05 level (2-tailed).

** Correlation is significant at the .01 level (2-tailed).

Finally, a subset of individuals consented to have their MindBody data shared with the research team to gather information on attendance. Participants (n = 92) were members for a range of less than 1 year to 8 years and attended 1 to 276 classes in the previous 12 months. Notably, 88% of this subset met physical activity recommendations according to their L-Cat response.

#### Correlations

##### Key psychosocial variables

Four significant moderate associations were detected: Self-compassion and perceived stress had a moderate negative correlation (*r* = −.31, *P* = .000); as self-compassion increased, perceived stress decreased. Self-compassion and mindfulness had a moderate positive correlation (*r* = .61, *P* = .000); as self-compassion increased, mindfulness increased. In addition, number of days attended was significantly associated with positive perceptions of cohesion and positive self-compassion scores (*P* = .000). Please see Table 2 for full correlation matrix.

### Qualitative

Across the 18 participants, the three most salient themes were: community, mental health benefits, and physical benefits. Most participants (n = 13, 72%) indicated that the community aspect was what brought them to practice at the yoga studio. Example meaning units from this theme included: “what really kept me coming was the sense of community” and “it feels like hOMe [tone inflection for OM].”

In addition, 50% (n = 9) of participants indicated that the mental health benefits of yoga were what led them to practice yoga at the partnering yoga studio. Meaning units for this theme included statements such as: “getting back mentally” and “it makes me really happy.”

The third major theme was the physical benefits of yoga. Meaning units for physical benefits were provided by 50% (n = 9) of the participants. The 2 subthemes of physical benefits were yoga as a form of exercise or workout or yoga to balance out the other activities they performed. Five of the 9 (55%) participants who mentioned physical health benefits used yoga as a workout as expressed through meaning units such as “the asana, the practice and how my body feels,” “and what I love, especially about, [is] being fit and strong,” and “I practice here for the . . . self- growth in my own practice physically.” The other 4 of the 9 individuals who mentioned physical health benefits (44%) provided meaning units for the subtheme related to yoga as a balance to other workout regimens such as: “but I really wanted to get some strength in my muscles that I can’t get from weight lifting,” “yoga is the one thing to get all soreness out and bring balance [back into their legs after running long distances],” and “I love this just kind [of] for a stretching and lengthening [after running and doing CrossFit].”

Minor emergent themes identified in this data set were mindfulness, the variety of classes offered at the yoga studio, the teachers employed by the yoga studio, the yoga studio itself, and the holistic health benefits that yoga can provide. In this study, 22% (n = 4) of the participants reported that the mindfulness aspects of yoga were what brought them to practice at the yoga studio. Meaning units for this theme included: “meditation,” “set my attention for the day, week, month . . . the yoga studio supports that for me,” and “it grounds me”. Related to the minor theme of mindfulness is the holistic health benefits of yoga that was reported by 6% (n = 1) of the participants. The meaning unit for this theme was “I personally do this [yoga] for holistic health.” The other 3 minor engagement themes presented in the data set for this study were related to student perceptions of the yoga studio itself.

Overall, 50% (n = 9) of the participants reported that they practice at the yoga studio because of the class structure. Twenty-two percent (n = 4) of the participants practiced at the yoga studio because of the yoga teachers who were employed by the studio owner. Meaning units for this theme included “the teachers are amazing,” and “the instructors are very well-versed and make it such an open atmosphere.” In addition, the nonjudgmental teaching style and the client-centered emphasis that instructors integrated into their classes were highlighted in the data set and counted as meaning units. For example, one participant mentioned that instructors incorporated certain poses to treat injuries. A minor theme related to the teachers employed at the yoga studio was the variety in yoga classes offered: 17% (n = 3) of participants reported that the many different yoga classes offered at the yoga studio was what brought them to practice there. Examples of meaning units included: “I can do yoga every day, at different times and [a] variety of different classes,” and “I like the different types of yoga that they [the yoga studio] offer.” Finally, 11% (n = 2) reported that they practiced at the yoga studio because of the environment or atmosphere of the studio. Meaning units that contributed to this minor theme included: “the relaxed atmosphere,” and “a place, an atmosphere to escape my actual responsibilities . . . provides me (with) knowledge.”

## Discussion

Limited research has been conducted within existing yoga studios and communities regarding psychosocial outcomes, physical activity behaviors, and yoga studio satisfaction. This study aimed to fill the gap in the literature around these 3 issues and found that yoga practitioners in this community-based studio have many positive perceptions (of yoga and the studio) as well as engage in positive health behaviors. Overall, the quantitative data suggest that yoga studio clients who participated in this study were moderately stressed, mindful, self-compassionate, and cohesive. However, only half of the participants were meeting physical activity recommendations. Self-compassion and perceived stress were negatively associated. This is unsurprising as self-compassion measures how individuals view themselves during times of stress. In addition, self-compassion is a type of mindfulness in which an individual perceives his or her situation without judgment, so the measured association between self-compassion and mindfulness, although not previously explored in community settings, is in the expected direction. Positive perceptions of the studio itself were evident in the high scores for all variables of interest (the environment, teachers, etc.), and these positive perceptions were also voiced by the individuals who shared testimonials as to why they practiced at this location. The qualitative data contribute to the extant literature by highlighting that, while people appreciated the mental and physical benefits of yoga as well as teachers and the structure, the practitioners were most likely to attend the studio due to the sense of community. While many of these relationships were in the predicted directions, there are several empirical and pragmatic observations to consider.

First, practitioners were rather cohesive and there was a significant positive correlation between the number of days individuals attend classes and positive perceptions of cohesion. Notably, this did not hold true for duration of membership and perceptions of cohesion. In other words, more frequent attendance was associated with higher perceptions of cohesion, but longer duration of membership was not associated with higher perceptions of cohesion. Simply having a membership did not provide the benefit of cohesion; individuals actually have to operationalize their membership through class attendance to experience cohesion. Relatedly, in a 1-year (6-month intervention, 6-month maintenance phase) stretching versus yoga intervention, preliminary results indicated that the stretching group significantly improved stress as measured by cortisol levels when compared to the yoga group.^26^ The researchers suggested that the key to this difference was that the stretching sessions were interpersonally dynamic whereas the yoga sessions did not prompt social interactions.^26^ This opportunity for engagement (i.e., facilitated by instructor, environment, or group norms) is the key of a “true group” rather than a group of individuals exercising at the same time.^27^

Therefore, there may be opportunities for low-dose group cohesion interventions at yoga studios. A group dynamics–based intervention features key principles of group environment, group structure, and group processes that may include interaction and communication, goal setting and progress updates, putting people in proximity to each other, and friendly competition.^28,29^ These principles are all represented in the studio’s 30-day challenge (described above) and may be a low-dose, high reward group dynamics–based intervention that may improve attendance, cohesion, inclusivity, and diversity. Future work is needed to explore the causal relationships between the 30-day challenge and outcomes of interest (attendance, cohesion, etc).

Our second observation is that tailored studio offerings may be beneficial to extend reach and representativeness. This aligns with other implementation research in that work is needed to understand for whom, under what conditions, and how a particular intervention or phenomenon works. For example, individuals from health disparate populations may need beginner level classes, high quality instructors, and explanation of yoga’s benefits. These key needs can be integrated into studio offerings or strategy but may not be impactful and well-received by those more familiar with yoga. Therefore, for individuals newer to the studio, an introductory series may be beneficial. In addition to building knowledge and self-efficacy in a yoga practice, this introductory series could also launch a cohort of new practitioners to feel more cohesive, which could lead to greater adherence.^30^ The studio has offered a 4-week introductory series four times and limits these sessions to 15 individuals (to ensure individualized feedback and community) for the past 2 years. Future work is needed determine the feasibility and acceptability of offering an introductory series, and the mental and physical health benefits that may result from participation.

While the participants in this study were reflective of nationwide yoga participants (e.g., predominantly Caucasian females)^8,13^ and the community in which the study was conducted, the studio values a focus on diversity and inclusivity. For example, a few community members requested a female-only yoga class. After careful consideration, studio management determined that a female-only class felt exclusive rather than inclusive. To meet the needs of these individuals, however, the studio owner and manager responded by offering a series for these women with special considerations addressed in that the class was led by a female and the windows were covered to accommodate cultural preferences (i.e., practitioners were Muslim). Another strategy to promote diversity in yoga participation (e.g., more diverse in terms of gender, race, ethnicity, religion, physical activities, and age) is to have more instructors from diverse backgrounds so that practitioners can identify themselves in their instructor.^31^ Future work is needed to understand what studio offerings may attract more individuals from diverse populations.

Finally, it is notable that half of the participants self-reported that they were meeting the physical activity guidelines for Americans. This may seem low considering that they engage in physical activity during a yoga class, but it is higher than the national average (e.g., 23.5% nationally vs 54% of studio members).^32^ Since the other half of the participants were not meeting recommendations, these results highlight opportunities to (1) test the physiological benefits of each yoga practice offered at this specific studio and (2) share these physiological benefits with practitioners at the studio. On the other hand, this low rate may also simply be reflective of the limitation that the L-Cat was not previously validated with this population. Future work is needed to determine the veracity of the L-Cat responses or to determine a better fit measure of physical activity behaviors of yoga practitioners.

Another area for future exploration is the fact that few participants (0.7%) reported attending the meditation-only classes despite stating that they attend yoga classes for mental health benefits. This may indicate that participants perceive receiving mental and physical health benefits in standard yoga-studio class practices, as opposed to needing to attend a stand-alone meditation class. Future work is needed to explore these relationships.

These preliminary data and potential implications for future directions are an important first step for this academic-studio partnership. However, this study has several limitations based on its design. First, as the survey portion was cross-sectional, it is impossible to make causal inferences between psychosocial factors and yoga class attendance or physical activity behaviors and yoga class attendance. Second, this study did not capture the confounding variables of yoga self-efficacy^33^ and yoga acceptability.^34^ Third, there may have been selection bias in the sampling (i.e., convenience sampling), resulting in systematic bias that leads to skewed results. This selection bias in the sampling might have been amplified by the availability of the survey during a community event where vegan snacks were offered at the studio; class attendees who chose to attend the event (and complete the survey) may systematically differ from class attendees who did not attend the event. Fourth, both internal and external validity might have been affected by the study setting (i.e., administering the yoga-based survey within a yoga studio during a social event): Internal validity might have been decreased by social desirability effects as participants may have depicted themselves as consistent with yogic stereotypes, especially since at least some participants completed the survey in close proximity to studio staff, teachers, and students. External validity might have been decreased by setting interaction (ecological validity), making it inappropriate to generalize findings to other locations, occasions, and times. Fifth, criterion validity of the L-Cat might have been reduced by ambiguity over how yoga classes (i.e., especially different types of yoga classes) fit into the physical activity categories, or by ambiguity over whether participants should include yoga classes at all in their self-reported physical activity. Sixth, the sociodemographic characteristics of yoga practitioners at this studio are in line with national data suggesting that yoga is most commonly practiced by Caucasian, college-educated females. In fact, the proportion of females in this study was slightly higher than in other studies of yoga in community settings.^8,35^ Future work is needed to identify if perceptions and behaviors within the studio vary by key demographic variables or if the same positive perceptions are observed in other community-based yoga studios.

Regardless of these limitations, this work provides an overview of the population, classes, and psychometric measurement of practitioners in a real-world setting. These data will be used to inform future decisions that will be empirically and practically meaningful.^36^
